# Plasmonic Au Array SERS Substrate with Optimized Thin Film Oxide Substrate Layer

**DOI:** 10.3390/ma11060942

**Published:** 2018-06-04

**Authors:** Zachary T. Brawley, Stephen J. Bauman, Ahmad A. Darweesh, Desalegn T. Debu, Faezeh Tork Ladani, Joseph B. Herzog

**Affiliations:** 1 Microelectronics-Photonics Graduate Program, University of Arkansas, 731 W. Dickson St., Fayetteville, Arkansas, AR 72701, USA; ztbrawle@email.uark.edu (Z.T.B.); sjbauman@email.uark.edu (S.J.B.); aadarwee@email.uark.edu (A.A.D.); 2 Department of Physics, University of Arkansas, 825 W. Dickson St., Fayetteville, Arkansas, AR 72701, USA; dtdebu@email.uark.edu (D.T.D.); ftorklad@uark.edu (F.T.L.)

**Keywords:** plasmonics, thin film, SERS, computational electromagnetics, nanowires, nano-optics, grating, array

## Abstract

This work studies the effect of a plasmonic array structure coupled with thin film oxide substrate layers on optical surface enhancement using a finite element method. Previous results have shown that as the nanowire spacing increases in the sub-100 nm range, enhancement decreases; however, this work improves upon previous results by extending the range above 100 nm. It also averages optical enhancement across the entire device surface rather than localized regions, which gives a more practical estimate of the sensor response. A significant finding is that in higher ranges, optical enhancement does not always decrease but instead has additional plasmonic modes at greater nanowire and spacing dimensions resonant with the period of the structure and the incident light wavelength, making it possible to optimize enhancement in more accessibly fabricated nanowire array structures. This work also studies surface enhancement to optimize the geometries of plasmonic wires and oxide substrate thickness. Periodic oscillations of surface enhancement are observed at specific oxide thicknesses. These results will help improve future research by providing optimized geometries for SERS molecular sensors.

## 1. Introduction

Surface enhanced Raman spectroscopy (SERS) molecular sensing has seen increased attention in recent years due to its ability to detect analyte molecules, even down to single molecule detection capabilities [1,2,3]. SERS can be used for a variety of chemical sensing including biological and inorganic molecules [4,5] and can be used with multiple material phases [6,7]. Because of this, SERS can be used in a variety of applications such as improved biomedical technologies, substance detection, and experimental chemical sensing [8,9,10]. Raman spectroscopy takes advantage of vibrational modes in analyte molecules, which weakly scatter light, to produce a characteristic spectrum with peaks corresponding to shifts in energy compared to the incident electromagnetic radiation; however, it is limited by the small signal strength produced by molecules.

Plasmonic nanoantennae can vastly improve the signal strength of molecules on SERS substrates by focusing incident light into ultra-small regions that enhance the electric near-field by many orders of magnitude [11,12,13]. In addition to enhancing the incident light, they can also couple to the Raman signal of the molecule and, therefore, enhance detection [14,15]. To do this, plasmonic nanogratings have been fabricated with geometries optimized in such a way as to produce the maximum possible electric field [16,17]. In turn, more intense electric fields will interact with individual molecules, producing a much greater scattering signal than for non-optimized geometries. Previous studies have analyzed the electric field in only specific regions of the structure or studied the reflectance/transmittance of the light [18,19]. To have a more complete picture of the SERS enhancement of nanograting structures, the near-field must be studied across the entire surface because molecules from which the signal is scattered do not reside solely in gap regions.

Computational modeling can be a useful tool for probing the near-field enhancement in extremely small regions prior to fabrication and experimental optical characterization. Current computational methods for probing plasmonic structures includes, but is not limited to, finite difference time domain (FDTD), discrete dipole approximation (DDA), and finite element method (FEM), the latter of which was the method used in this study [20,21,22]. Line averages across the surface of the substrate were used to gain a better insight into the electric field enhancement across the entire surface as opposed to the integration regions studied in previous papers [18,23]. For this structure to demonstrate practical SERS biosensor molecular detection capabilities, it must be able to sense molecules in regions that are not only between the nanowires, where the highest enhancement occurs, but also spread out across the entire surface of the device. This work helps to analyze a greater active sensing area by exploring signal enhancement in an increased detection region.

Furthermore, many papers study plasmonic enhancement in sub-100 nm regions due to optimized enhancement occurring below this gap width threshold [24,25,26,27]. However, additional peaks in enhancement have been observed above 100 nm geometries resonant with the wavelength of the light and period of the structure [28,29,30]. This work shows that, though these higher geometric modes are weaker in magnitude, they can still yield significant enhancement values for improved sensing capabilities. In addition, the thickness of a silicon dioxide thin film substrate is studied to find resonant thicknesses that further improve the enhancement capabilities of the structure; this optimizes light on the surface of the substrate, where the molecules of interest are located during SERS. A similar technique has been used to optimize contrast in graphene studies [31,32]. Devices are then proposed for improved plasmonic surface enhancement both at optimized geometries and at resonant modes with larger structures/gaps. With larger gap and wire geometries providing significant enhancement, reliance on advanced two-step nanogap or nanoslit lithographic techniques can be eliminated [18,33,34,35]. While there are many nice techniques for obtaining quality SERS substrates [36,37], this work highlights that there are some interesting optical features in patterned nanograting structures that have not been fully explored. Specifically, this work can improve fabrication efficiency by allowing for standard nanofabrication techniques such as electron-beam lithography (EBL), photolithography, or chemical self-alignment [38,39,40,41] instead of using advanced methods to fabricate sub-10 nm gaps [33,42,43]. As a result, SERS sensors can more easily be designed and built in industry as commercially viable products.

## 2. Materials and Methods

**Figure 1:**
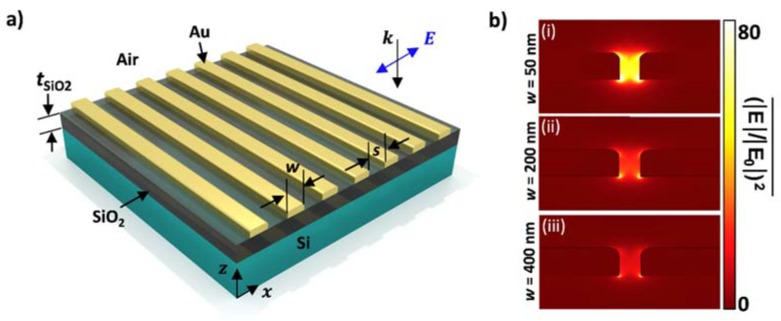
Depiction of the modeled grating of Au plasmonic nanowires bonded to a SiO_2_ thin film by a Ti adhesion layer atop a Si substrate. (**a**) Shows a schematic of the simulated structure under the presence of *λ*_0_ = 785 nm light incident normal to the surface and polarized in the *x*-direction, perpendicular to the length of the nanowires. The parameters swept in this study were wire width (*w*), electrode spacing (*s*), and SiO_2_ thickness (*t*_SiO_2__); (**b**) Optical enhancement hotspots in 2D cross-sections of structures modeled as infinite wires with *t*_SiO_2__ = 330 nm, *s* = 10 nm, and (i) optimized value at *w* = 10 nm, (ii) median value at *w* = 200 nm, and (iii) minimum value at *w* = 400 nm.

A finite element method [44] was used to study two-dimensional cross sections of Au plasmonic nanowires. A line average of all mesh point values along the surface of the structure was used to calculate optical enhancement. This enhancement is defined as the absolute value of the magnitude of the localized electric near-field (|E|) divided by the absolute value the incident electric far-field $\left(\left|E_{0}\right|\right)$, all squared, which is proportional to the light intensity [45,46]. This study improves previous work by calculating the line average of ${\left(\left|E\right|/\left|E_{0}\right|\right)}^{2}$ across the entire structure instead of only integrating in the gap region previously studied, which does not give a full view of the enhancement across the system’s entire surface [18]. Incident light of constant wavelength *λ*_0_ = 785 nm, a common probe laser wavelength used for Raman spectroscopy, was simulated as polarized in the *x*-direction, which was perpendicular to the length of the nanowires, and incident normal to the surface of the structure in the *z*-direction, as seen in Figure 1a. Complex optical material properties of Au, Ti, SiO_2_, and Si were used, and the top surrounding space was simulated as air [47,48,49]. Fillets with a radius of 4 nm were used on the upper corners of the nanowires to model the geometry of real fabricated devices instead of using a perfectly rectangular cross-section.

Parametric sweeps were performed for variables including the grating structure width (*w*), spacing (*s*), and silicon dioxide thickness (*t*_SiO_2__) as labeled in Figure 1a in order to optimize surface enhancement. The silicon substrate was simulated to be infinitely thick using ports to eliminate back-scattering from the bottom boundary of the model. The period of the structure, defined as *P* = *w* + *s* was modeled as an infinite array using periodic boundary conditions, and the lengths of the wires were approximated as infinite. Previous work has demonstrated that Ti can be used as an adhesion layer to bond Au to SiO_2_ [50]. In this study, a Ti thickness of 1 nm was used so as to minimize any plasmonic losses, which occur as the Ti thickness increases [50]. This loss effect due to Ti thickness occurs because Ti is a weak plasmonic material, which has very large imaginary permittivity values compared to Au; therefore, it absorbs plasmonic energy. The Au thickness was held constant at 15 nm.

Figure 1b shows localized electric field distributions in cross sections of Figure 1a. Here, three different geometries of wires of width (i) *w* = 50 nm, (ii) *w* = 200 nm, and (iii) *w* = 400 nm were used, and the gap spacing was constant at *s* = 10 nm so as to optimize field enhancement in the gap region as has been demonstrated in bow-tie structures for SERS devices [51] with standard nanofabrication techniques. Silicon dioxide thickness was held constant at *t*_SiO_2__ = 330 nm. It is evident in this figure that the localized electric field was greatest in the nanowire gap and largely influenced by the geometry of the structures. The field enhancement can be tuned to be up to 80 times higher than non-optimized geometries.

## 3. Results

The following section analyzes the parametric sweeps to gain a deeper understanding of where plasmonic-resonant electric field modes occur and optimizes *w*, *s*, and *t*_SiO2_ for the greatest near-field line average enhancement. Using these geometries, suggestions will be made on how to fabricate the most effective SERS molecular sensor using plasmonic devices.

### 3.1. Electrode Spacing Optimization

**Figure 2:**
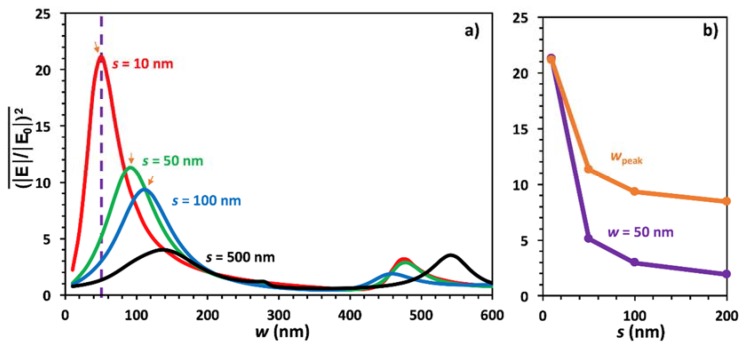
(**a**) Plot of line average enhancement versus *w* at *s* = 10 nm, 50 nm, 100 nm, and 500 nm; (**b**) plots the enhancement values from the peaks in (**a**) at different spacing values, *w*_peak_; and plots the enhancement value for a constant width, *w* = 50 nm, for spacing values in (**a**). The vertical purple dashed line in (**a**) illustrates from where the data for *w* = 50 nm in (**b**) is obtained, and the orange arrows show points used for *w*_peak_. (**b**) Further, plot includes an additional data point not shown in (**a**) at *s* = 200 nm.

The first simulation was conducted to build upon previous work by assuming that as *s* increases, optical enhancement decreases, but here a line average was taken across the entire surface as opposed to averaging over mesh points in specific regions [18]. Also, this work explored gap ranges greater than 100 nm, which has not been commonly studied using plasmonic nanowires for SERS. Electrode spacing was held constant at *s* = 10 nm, 50 nm, 100 nm, and 500 nm, and the wire width was swept from 10 nm to 600 nm in 10 nm increments at each value of *s*. The oxide thickness, *t*_SiO2_, was held constant at 330 nm because it has been shown that this thickness optimizes the plasmonic enhancement due to thin film effects [18]. The results of the sweep are shown in Figure 2. For the *s* values shown here, the peak values in the line average of ${\left(\left|E\right|/\left|E_{0}\right|\right)}^{2}$ decreased with increasing electrode spacing but only for *w* < 400 nm. There was also a shift in peak enhancement values towards larger wire widths with increasing *s*, appearing to demonstrate resonance with the period of the grating structure. One implication of this is that it is beneficial to keep *s* as small as possible when fabricating such grating structures in order to optimize the response by using the corresponding best *w*. However, there were additional plasmonic modes that are less in magnitude at higher *w* values (>400 nm) that were resonant, albeit more weakly, with the grating period, similar to previous modal period-versus-wavelength studies [29,30,52]. Figure 1b plots both *w* = 50 nm, the optimized wire width, and peak (*w*_peak_) enhancement values against *s* from 10 to 200 nm. The enhancement values of *w*_peak_ decreased exponentially with increasing *s*, as does the enhancement at constant width, *w* = 50 nm.

### 3.2. Thin Film Thickness and Electrode Optimization

**Figure 3:**
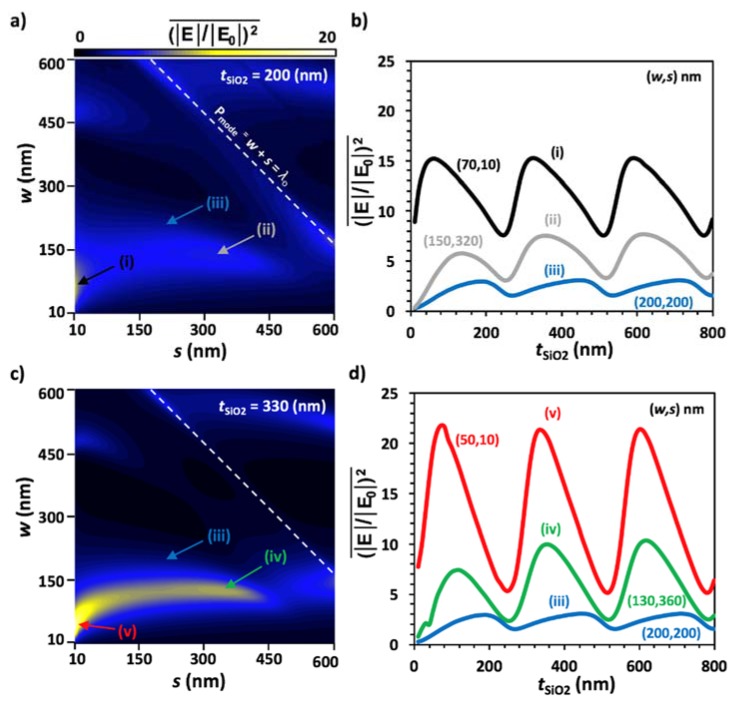
(**a**) Color plot of line average enhancement versus s and *w* with *t*_SiO_2__ = 200 nm. The arrows point to (i) optimized geometry values *w* = 70 nm and *s* = 10 nm, (ii) secondary peak geometry values *w* = 150 nm and *s* = 320 nm, and (iii) standard fabrication geometries *w* = 200 nm and *s* = 200 nm. The white dotted line indicates a resonant mode corresponding to the period of the structure at 785 nm, the same as *λ*_0_; (**b**) Sweep of line average enhancement versus *t*_SiO_2__ at (i), (ii), and (iii); (**c**) Second iteration of (**a**) with maximum *t*_SiO_2__ = 330 nm found from (**b**). The arrows point to (v) the optimized enhancement value occurring at *w* = 50 nm and *s* = 10 nm, (iv) secondary peak geometry values *w* = 130 nm and *s* = 360 nm, and (iii) standard fabrication geometries *w* = 200 nm and *s* = 200 nm; (**d**) Second sweep of line average enhancement versus t_SiO_2__ at (iii), (iv), and (v) to optimize *t*_SiO_2__. The geometry is optimized at *t*_SiO2_ = 50, 330, or 590 nm, *w* = 50 nm and *s* = 10 nm; however, a second peak occurs at *t*_SiO_2__ = 630 nm, *w* = 130 nm and *s* = 360 nm, which is more easily fabricated.

Figure 3 shows two iterations during the optimization of surface enhancement by varying *t*_SiO2_, *w*, and *s*. Figure 3a presents a color plot of a sweep of *s* and *w* from 10 nm to 600 nm in 10 nm steps, while *t*_SiO2_ was held constant at 200 nm as a baseline oxide thickness that would later be optimized. The color bar shows a gradient of enhancement from black to white representing low to high enhancement, respectively. The highest enhancement values occurred at (i), *w* = 70 nm and *s* = 10 nm. This is expected for plasmonic structures, with the greatest enhancement occurring at sub-100 nm geometries. What is exciting about these results, however, is that there were additional modes at greater *w* and *s* values. Arrow (ii) points towards a region of enhancement at *w* = 150 nm and *s* = 320 nm that corresponded to the second region with high enhancement values. These values were lower compared to (i), but the importance here was that they allow for more accessibly fabricated devices. It is also interesting that ${\left(\left|E\right|/\left|E_{0}\right|\right)}^{2}$ did not always decrease with increasing electrode spacing. There are additional plasmonic modes resonant with the geometry of the structure at greater *s* and *w* values. Another mode corresponds to the period of the structure equal to the wavelength of incident light (*P*_mode_ = *λ*_0_), shown as the white dashed line with a slope of −1 on the color plot, which means that it occurred at a constant period [53]. It also allows for a range of geometries, so fabrication does not have to be as precise, but can deviate by up to ± 10 nm. The (iii) arrow shows a geometric combination with standard fabrication capabilities, *w* = 200 nm and *s* = 200 nm, for comparison.

Figure 3b shows the result of a sweep to optimize *t*_SiO_2__ from 10 to 800 nm with *w* and *s* set at (i), (ii), and (iii). It shows that there are periodic regions of constructive and destructive interference at the surface of the substrate, which yield high or low reflection, respectively, dependent on the wavelength of the light and the *t*_SiO_2__ height [54]. The thin film is optimized at *w* = 70, *s* = 10, and *t*_SiO_2__ = 330 nm; however, other oxide layers, *t*_SiO_2__ = 60 or 590 nm, etc., may be used with little to no loss in light enhancement. A slight shift in peak enhancement values was observed based on the nanowire surface geometry due to plasmonic surface effects and the period of the structure. The peak enhancement resulting from the parameters for (ii) were roughly half of the peak values for the optimized structure, still demonstrating a high sensing capability for applications which may benefit from relaxed fabrication constraints.

Figure 3c is a second iteration of Figure 3a but demonstrates the effect of using the optimal *t*_SiO2_ at 330 nm. It can be observed in the color plot that the enhancement greatly increased for the same ranges of *w* and *s* compared to that in Figure 3a. This indicates that the enhancement values were much higher at optimized oxide substrate thicknesses, which is expected. Also, the maximum enhancement values slightly shifted from (i) *w* = 70 nm, *s* = 10 nm and (ii) *w* = 150 nm, *s* = 320 nm to (v) *w* = 50 nm, *s* = 10 nm and (iv) *w* = 130 nm, *s* = 360 nm. On this iteration, it was necessary to sweep *t*_SiO_2__ once more to ensure that the structure was optimized, and the results are shown in Figure 3d. Although the peak ${\left(\left|E\right|/\left|E_{0}\right|\right)}^{2}$ values rose from 15 to a maximum of 22, the *t*_SiO_2__ values at which these peaks occurred did not shift. This indicates that the structure was, in fact, optimized. Were another iteration to be conducted, it would yield the same results because peak oxide thicknesses did not shift. Line averaged enhancement is optimized at *t*_SiO_2__ = 60, 330, or 590 nm, *w* = 50 nm and *s* = 10 nm; however, a second peak occurred at *t*_SiO_2__ = 630 nm, *w* = 130 nm and *s* = 360 nm. This geometric combination is more easily fabricated and still provides roughly half of the enhancement value of the fully optimized geometry.

## 4. Discussion

A major implication of this work is that enhancement values do not always decay at greater geometric values, as was previously assumed, but instead increase at specific *w* and *s* values resonant with the periodicity of the structure. This is, in large part, due to values which yield the largest constructive and deconstructive interference at the surface of the substrate. At specific periods, the polarized light passing through the surface will be reflected off the silicon dioxide layer boundary and constructively interfere, coupling with the light already incident from the top of the structure. In this case, one such resonant period happened to be equal to the wavelength of the light, which is dictated by the Rayleigh scattering anomaly, a grating phenomenon [55,56]. This caused an enhanced electric field at the surface, which led to an increased strength of localized plasmon resonances across the device surface. These effects can yield up to 22 times the optical enhancement of non-optimized structures.

The results can be compared to previous work [18,50] to understand how taking a line average across the surface of the SERS device compares to studying an integration area only over the gap regions. Bauman et al. studied a similar structure while only looking at an area around the gap region [18] and showed an enhancement value of around 67 for a similar structure but with a 5 nm spacing, as compared to the value of 22 obtained in our work using a line average and a spacing of 10 nm. If we were to look at a smaller spacing this enhancement value would almost double, bringing it closer to, but still less than, the value obtained using a volume average [18,50]. This makes sense because the greatest enhancement values occur in the gap regions, so integrating only around the gap would yield higher results than integrating across the entire surface where much lower enhancement values are found. In [18], for larger widths, the secondary peak is greater in the gap compared to our surface-average study. The secondary peak, seen around 500 nm in Figure 2a, was much weaker than the initial peak at 50 nm in contrast to the relative peak amplitudes for gap regions. Again, this makes sense because larger wire widths lead to a greater space between gap regions, decreasing the overall surface-averaged result. So, the results of this paper are more realistic because molecules will be spread over the entire surface as opposed to very specific localized regions. Thus, this study is significant because even with entire line averages across the surface, enhancement can be up to 22 times that of non-optimized structures, showing that these results should be used when fabricating SERS devices for molecular sensing. Also, it is important to optimize geometries at smaller widths when possible and not around any secondary peaks.

Additional modes were also observed at higher *w* and *s* values due to the period of the structure causing increased reflection off the bottom silicon layer, as shown in Figure 2 and Figure 3. This allows for devices to be fabricated with more standard techniques such as electron-beam lithography, which eliminates the need to use more advanced techniques such as nanomasking [33]. Such advanced methods are required to make the smallest optimized structure or gaps below the typical resolution limits of optical or e-beam lithography. Although the devices may not generate the greatest enhancement possible, they are still highly efficient, causing optical enhancement values of up to 11 times greater than for non-optimized devices.

The standard equation for thin film interference, *t*_SiO_2__ = (2*πm*_d_ − *φ*_1_ − *φ*_2_)*λ*_0_/(4*πn*), can be used to determine which oxide thicknesses will result in deconstructive interference shown in Figure 3d, where *m*_d_ = {0, 1, 2, 3, …}, λ_0_ is the incident wavelength, *n* is the refractive index of SiO_2_ at *λ*_0_ = 785 nm, and *φ*_1_ and *φ*_2_ are the phase retardations of the upper and lower boundaries of the thin film [57]. Here, *φ*_1_ = *φ*_2_ = 0 when light is incident normal to the surface in the *z*-direction. On the other hand, the maximum values cannot be precisely determined from this standard thin film interference equation since the maximum surface enhancement values are not at the exact midpoint between the minimum (deconstructive) thickness values; this highlights the importance of this work. Here, we show that in order to optimize the thin film interference effects for constructive interference to maximize surface enhancement, one should use an oxide thickness that roughly corresponds to *m*_c_ = *m*_d_ + 1/3. This shift from the midpoint value comes about since we calculated the average surface enhancement in the near-field and not the total reflection in the far-field, which is what the standard thin film equation does. Further, the plasmonic modes that scattered and resonated with the incident and reflected light altered the total surface enhancement as well. Therefore, the maximum surface enhancement for a plasmonic grating on a thin film oxide will not necessarily occur for the thickness value exactly halfway between two adjacent thicknesses giving destructive interference, but instead are at a point roughly 1/3 of the way toward the lower deconstructive interference thickness for optimized geometry values.

If this were purely a structure with a single thin film layer, then the simple thin film equation could be used to predict the peak position. However, introducing a periodic plasmonic structure on the surface effectively distorts the reflection phase factors, *φ*_1_ and *φ*_2_, which caused a shift in this peak position. There were multiple interfaces at play here (Air/Au, Au/Ti, Ti/SiO_2_, Air/SiO_2_, SiO_2_/Si), and a variation of these interfaces across the entire surface where we had the wires and the spaces; therefore, a simple thin film equation is no longer appropriate, but instead the periodic variations on the surface need to be considered [57]. Finally, it is important to note that here we calculated near-field surface enhancement, not far-field reflection, so these simulations were necessary to predict the precise peak shift instead of a thin-film interference equation.

**Figure 4:**
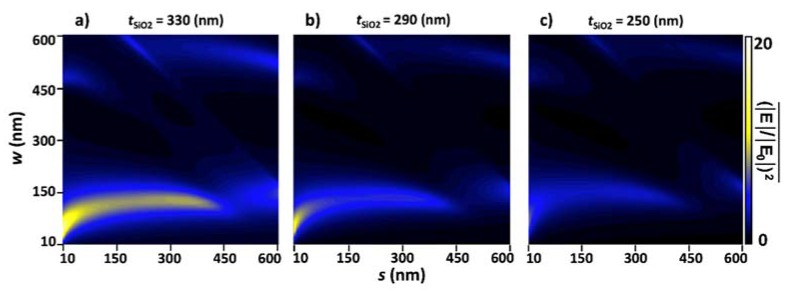
Color plot of line average enhancement versus *s* and *w* at three *t*_SiO_2__ values. (**a**) Maximized enhancement at *t*_SiO2_ = 330 nm, (**b**) median at *t*_SiO_2__ = 290 nm, and (**c**) minimized at *t*_SiO_2__ = 250 nm.

Figure 4 shows three color plots of parametric sweeps of *w* and *s* from 10 nm to 600 nm with three different *t*_SiO_2__ values. Figure 4a–c are plots of maximum, median, and minimum enhancement values from the thin film oxide layer, *t*_SiO_2__, in accordance with Figure 3d. The same color plots would have been produced for additional resonant modes as well, such as *t*_SiO_2__ = 590 nm, 560 nm, and 520 nm. It is observed that, when plotted on the same color range, there was a significant difference in average ${\left(\left|E\right|/\left|E_{0}\right|\right)}^{2}$ for each substrate thickness; therefore, it is important to use very specific substrate thicknesses when fabricating. Low deviation in substrate thickness was even more significant than wire dimensions, as there is a higher peak enhancement gradient from *t*_SiO2_ than from surface geometries.

## 5. Conclusions

Plasmonic structures have been shown to improve SERS sensors by focusing light into extremely small gap regions where analyte molecules are located, thus enhancing the signal produced by the molecules, making them more easily detectable. Previous results have shown that optical enhancement in gap regions increases as the geometries (width and spacing) of the grating structure decrease. Although this work draws the same conclusions for an optimized structure, it extended the analyzed size regime above 100 nm, and enhancement across the entire surface of the sample was considered versus calculating in localized integration regions. Additional plasmonic modes were observed at higher geometries greater than 100 nm, which had peak enhancement values roughly half of the values produced by optimized structures but still 11 times greater than those produced by non-optimized geometries. Average enhancement also oscillated periodically with resonant peaks of constructive and deconstructive interference based on the thickness of a silicon dioxide thin film atop a silicon layer. These results were verified by thin film interference theory.

The nanowires and thin film were fully optimized at *t*_SiO_2__ = 60, 330, or 590 nm, *w* = 50 nm and *s* = 10 nm; however, a second resonant peak occurred at *t*_SiO_2__ = 630 nm, *w* = 130 nm and *s* = 360 nm that was more accessible in practice through standard fabrication techniques such as EBL or high-resolution photolithography. So in addition to optimizing the patterned structure, it is also important to optimize the oxide layer since this can affect the results by a factor of 4. These results can help improve the time and efficiency needed to fabricate plasmonic-based SERS devices, as compared with sub-10 nm gap two-step lithography techniques.
